# Effects of the soil microbiome on the demography of two annual prairie plants

**DOI:** 10.1002/ece3.6341

**Published:** 2020-06-14

**Authors:** Hannah S. Reynolds, Rebekah Wagner, Guangzhou Wang, Haley M. Burrill, James D. Bever, Helen M. Alexander

**Affiliations:** ^1^ Department of Ecology & Evolutionary Biology University of Kansas Lawrence KS USA; ^2^ Kansas Biological Survey University of Kansas Lawrence KS USA

**Keywords:** annuals, arbuscular mycorrhizal fungi, demography, mutualist, pathogen, prairie, soil microbiome

## Abstract

1. Both mutualistic and pathogenic soil microbes are known to play important roles in shaping the fitness of plants, likely affecting plants at different life cycle stages.
2. In order to investigate the differential effects of native soil mutualists and pathogens on plant fitness, we compared survival and reproduction of two annual tallgrass prairie plant species (*Chamaecrista fasciculata* and *Coreopsis tinctoria*) in a field study using 3 soil inocula treatments containing different compositions of microbes. The soil inocula types included fresh native whole soil taken from a remnant prairie containing both native mutualists and pathogens, soil enhanced with arbuscular mycorrhizal (AM) fungi derived from remnant prairies, and uninoculated controls.
3. For both species, plants inoculated with native prairie AM fungi performed much better than those in uninoculated soil for all parts of the life cycle. Plants in the native whole prairie soil were either generally similar to plants in the uninoculated soil or had slightly higher survival or reproduction.
4. Overall, these results suggest that native prairie AM fungi can have important positive effects on the fitness of early successional plants. As inclusion of prairie AM fungi and pathogens decreased plant fitness relative to prairie AM fungi alone, we expect that native pathogens also can have large effects on fitness of these annuals. Our findings support the use of AM fungi to enhance plant establishment in prairie restorations.

## Graphical abstract

Composition of the soil microbiome is important to the fitness and success of annual prairie plants. In a field experiment, plants inoculated with native prairie arbuscular mycorrhizal fungi (AMF) perform better than plants inoculated with native prairie soil (containing both native AMF mutualists and native pathogens) and uninoculated plants. This research suggests that native prairie AMF mutualists can have important positive effects on the fitness of early successional native plants, and supports the use of mycorrhizal fungi to enhance native plant establishment in restoration environments.

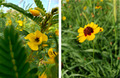

## 1 INTRODUCTION

Experimental and theoretical studies suggest that soil microbes play a central role in plant species coexistence and community structure (Bever, Mangan, & Alexander, 2015; Burns, Brandt, Murphy, Kaczowka, & Burke, 2017; Mangan, Herre, & Bever, 2010; Thrall, Bever, Mihail, & Alexander, 1997). Increasing evidence also indicates that soil microbes are important in native plant restoration efforts (Koziol et al., 2018). For microbes to have these roles, they must have an effect on plant fitness, the contribution of an individual (in terms of offspring) to the next generation. Complete studies of microbial effects on fitness should integrate survival and reproduction at different stages of the host organism's life (Antonovics & Alexander, 1989; Malmstrom & Alexander, 2016). Such studies are rare and are not trivial given that the soil microbiome includes diverse organisms and both mutualists and pathogens.

Microbes that are often mutualistic include types of nitrogen‐fixing bacteria (*Rhizobium*, *Azospirillum*) and arbuscular mycorrhizal (AM) fungi. The latter group of fungi form particularly important associations with the roots of host plants, providing water and phosphorous in exchange for sugars produced by the plant and potentially providing resistance to pathogens (Smith & Read, 2008). By supplying the plant with these benefits, AM fungi can greatly increase plant growth (Hoeksema et al., 2018; Koziol & Bever, 2015). AM fungal species are, however, not always mutualists. The degree to which a particular plant–AM fungal interaction is mutualistic or pathogenic depends on the plant species, AM fungal species, and abiotic factors such as soil nutrient and water availability (Cheeke, Zheng, Koziol, Gurholt, & Bever, 2019; Hoeksema et al., 2018; Johnson, Graham, & Smith, 1997; Jones & Smith, 2004). Most work on plant–AM fungal interactions has focused on agronomic plants; there have been fewer studies of the effects of AM fungi on demography of wild plants. Notably, work by Koide and coauthors has demonstrated that the presence of AM fungi can have large positive effects on plant reproduction, seed size, and germination of *Abutilon theoprasti* (Koide & Dickie, 2002; Stanley, Koide, & Shumway, 1993). While the identity of AM fungal species has been shown to have large effects on plant growth (Cheeke et al., 2019; Koziol & Bever, 2016), less is known on the importance of AM fungal species composition on elements of plant demography.

Soil pathogens also have diverse effects (Ampt, van Ruijven, Raaijmakers, Termorshuizen, & Mommer, 2019; Jarosz & Davelos, 1995; Thrall et al., 1997). For example, infection by *Pythium* can lead to increased seed and seedling mortality and decreased seedling growth (Alexander & Mihail, 2000; Hendrix & Campbell, 1973; Mills & Bever, 1998). In India, a build‐up of a soil‐borne fungus, *Fusarium semitectum,* under an introduced weedy plant resulted in suppression of plant height in native and naturalized plants (Mangla & Callaway, 2007). Much of our inference on the importance of pathogens in plant community structure comes from plant–soil feedback experiments (Bever, Westover, & Antonovics, 1997; Crawford et al., 2019), which have emphasized short assays (e.g., microbe effects on plant growth) as proxies for fitness. Notable exceptions include work by Burns et al. (2017) and Dudenhöffer, Ebeling, Klein, and Wagg (2018), who have focused on entire plant life cycles, including effects of soil microbes on plant reproduction. Miller, Perron, and Collins (2019) have studied feedback at the seed stage.

The demographic effects of microbes on plants are relevant in the tallgrass prairie. For thousands of years, prairies covered much of central North America but this ecosystem was largely lost with EuroAmerican settlement in the mid‐1800s and subsequent agricultural use (Samson & Knopf, 1994). Extensive plowing of the soil, as well as chemical treatments, led to postagricultural soils with a degraded soil biotic community (Oehl et al., 2003). Anthropogenic disturbance can favor weedy AM fungal species (House & Bever, 2018). However, small never‐plowed remnant prairies do occur with diverse plant and microbial species. Reintroduction of these native prairie soil microbes, through either inoculation with AM fungi or whole‐soil additions, can be important in establishing prairie plants in restoration sites in degraded postagricultural soils (Koziol et al., 2018; Lubin, Schultz, Bever, & Alexander, 2019; Middleton & Bever, 2012). Late successional native plant species have been found to be sensitive to AM fungal species composition and particularly benefit from reintroduction of native AM fungi (Cheeke et al., 2019; House & Bever, 2019; Koziol & Bever, 2016, 2017; Middleton et al., 2015).

Most work on native prairie soil communities has focused on AM fungi. The extent to which loss of native prairie pathogens might affect the establishment of native plants following agricultural disturbance is not known. However, in studies where both early and late successional prairie plants were inoculated with whole prairie soil, there were positive effects of soil addition on plant growth for only the late successional plants (Middleton & Bever, 2012). Native soil pathogens may have disproportionally affected the performance of early successional plants. Indeed, early successional plants appear to be more susceptible to pathogen effects than are their late successional counterparts, perhaps due to the differences in their life history (Bauer, Mack, & Bever, 2015; Herms & Mattson, 1992). Early successional species, including many annuals with rapid growth, may not allocate as much energy to pathogen resistance as long‐lived later successional plants (that may need pathogen resistance in order to survive long enough to reproduce). Due to these differences between early and late successional plants, pathogen build‐up in the soil microbiome may be a driver of ecological succession (Bauer et al., 2015; Kardol, Bezemer, & van der Putten, 2006; Van der Putten, Vandijk, & Peters, 1993). As pathogens accumulate in the soil, early successional plant populations are diminished, and mid‐ and late successional plants can begin to grow.

We examined the effects of the soil microbial community on the fitness of two species of annual tallgrass prairie plants. Specifically, we measured both plant survival and reproduction in a field study where plant soil was manipulated. We focused on three soil treatments: native whole prairie soil (never‐plowed remnant prairie soil containing both mutualists and pathogens; hereafter, native whole soil), soil enhanced with only native prairie AM fungi (hereafter, native AM fungi‐enhanced soil), and uninoculated postagricultural soil. A priori, we expected that plants grown with (a) native AM fungi‐enhanced soil to perform better than those in postagricultural soil if AM fungi are acting as a mutualist in the former soil, (b) native AM fungi‐enhanced soil to perform better than those in the native whole soil if negative effects of the pathogenic component of the native soil community are greater than positive effects of AM fungi, and (c) the native whole soil to perform better than those in postagricultural soil if the mutualistic component of the community is most prevalent (and the opposite to occur if pathogens dominate the interactions).

## 2 METHODS

### 2.1 Study organisms

**FIGURE 1:**
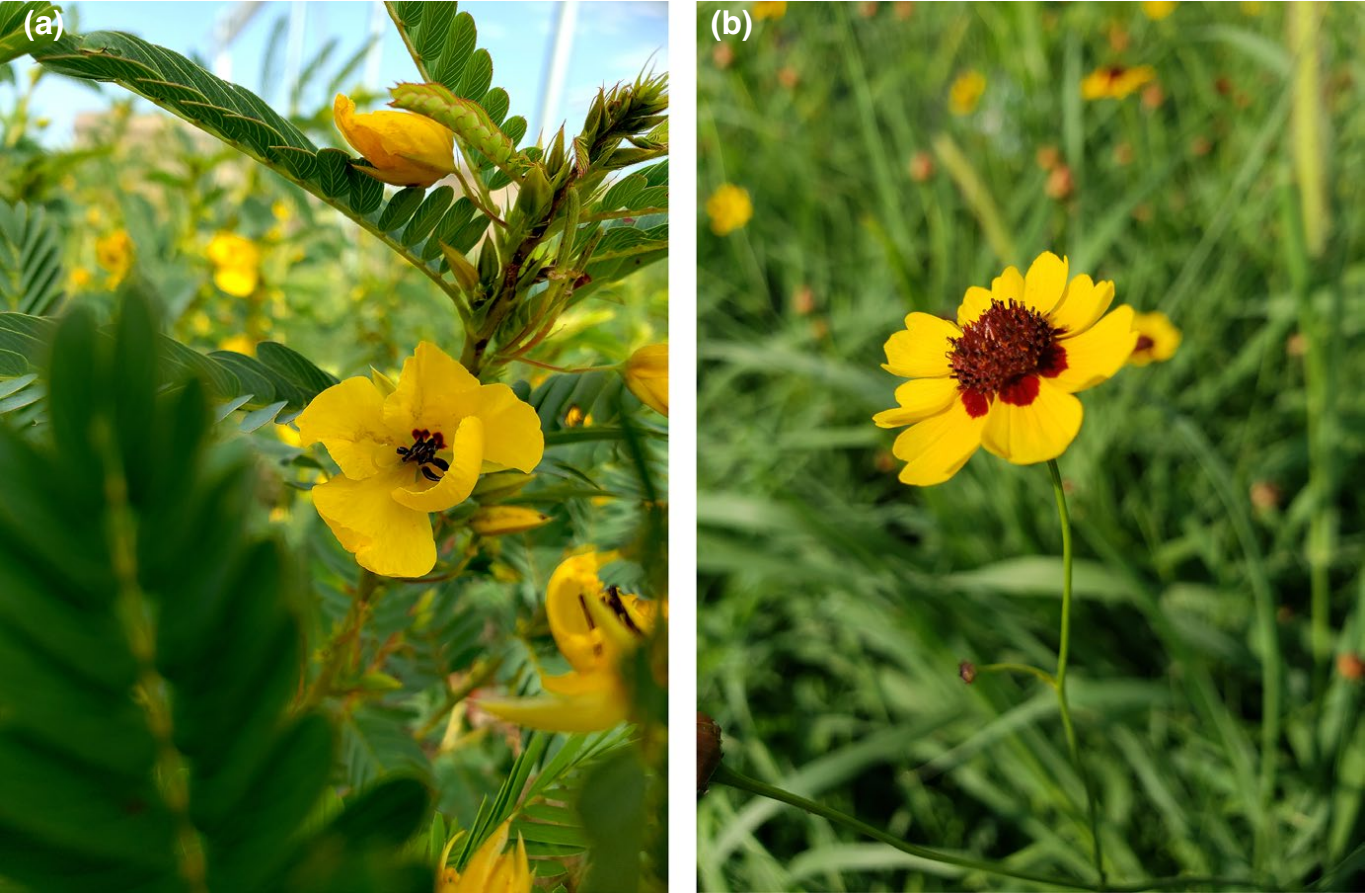
Flowering plants of (a) *Chamaecrista fasciculata* and (b) *Coreopsis tinctoria*. Both species are annual plants of the tallgrass prairie

*Chamaecrista fasciculata* (Fabaceae, “showy partridge pea,” Figure 1a) is an annual species commonly found in central and eastern North America (Kartesz, 2015). Mature plants typically grow to heights ranging from 0.15 to 1 m and are found in prairies, old fields, and roadsides (Haddock, Freeman, & Bare, 2015). This species has been well studied from an ecological and evolutionary perspective, including geographic patterns of genetic variation (Etterson, 2004) and root interactions with nitrogen‐fixing bacteria (Keller & Lau, 2018). For the latter, *Chamaecrista* associates with the genus *Bradyrhizobium* (Parker & Kennedy, 2006; Tlusty, Grossman, & Graham, 2004).

*Coreopsis tinctoria* (Asteraceae, “plains coreopsis,” Figure 1b) is found across central North America (Kartesz, 2015). This annual species is found in prairies, floodplains, and moist disturbed sites and reaches heights of 0.6–1.2 m (Haddock et al., 2015). Inflorescences consist of capitula with many small individual flowers. For simplicity of referring to the same terms for both plant species, we refer to capitula of *Coreopsis tinctoria* as “flowers.” From here onwards, we refer to both plant species by their generic names.

Both species have low coefficients of conservatism (*Coreopsis* = 1, *Chamaecrista* = 2) (Freeman, 2014). This scale ranges from 0 to 10; these low values are consistent with the annual plants' ability to establish themselves in recently disturbed areas; later successional perennial plants typically have higher scores (Herman et al., 1997). Consistent with these coefficients, *Coreopsis* was one of the first species to colonize areas near forb‐sown prairie restoration plots (Jaksetic, Foster, Bever, Schwarting, & Alexander, 2018) and it is a poor competitor with perennial grasses (Elliot & Van Auken, 2014). Similarly, *Chamaecrista* is found primarily in disturbed areas, including recently burned or animal‐disturbed parts of prairies and roadside habitat (Galloway & Fenster, 2000). Native AM fungi are less likely to benefit the growth of plant species with low coefficients of conservatism (Bauer, Koziol, & Bever, 2018; Koziol & Bever, 2015); such plant species are also less sensitive to AM fungal identity (Cheeke et al., 2019; Koziol & Bever, 2016).

### 2.2 Inocula and seedling establishment

#### 2.2.1 Soil inocula

We had three soil inocula treatments: native whole soil, native AM fungi enhanced, and uninoculated. The prairie soil used for the native whole inoculum was taken from a portion of the Anderson County Prairie Preserve which was to be destroyed for road development in eastern Kansas, USA (near Welda, Kansas 38.179600, −95.265695). Soil was collected during the week of 2 February 2018 and transferred to the University of Kansas Field Station (near Lawrence, Kansas; 39.050972, −95.191845) for use in this experiment. This remnant tallgrass prairie had been previously grazed, but not plowed. The uninoculated soil was produced by autoclaving prairie soil in a steamer cart for approximately 2.5 hr at a temperature of 76°C. The steaming process was done twice for all of the uninoculated soil in this experiment. Although the soil for this treatment was sterilized in the early stages of the study, we refer to this treatment as “uninoculated” as opposed to “sterile” due to the fact that all *Chamaecrista* seedlings had rhizobia additions and all seedlings were planted into a postagricultural field environment, and thus experienced a full set of microbes. For the native AM fungi‐enhanced soil, AM fungi taken from prairie remnants were added to autoclaved soil. This soil was created with a 1:2 ratio of native AM fungal inocula to autoclaved prairie soil. See Koziol et al. (2018) for details of AM fungal culture methods and Appendix 1 for more details of remnant prairie soil collection and AM fungal inocula species.

#### 2.2.2 Seedling establishment

Seeds were purchased from Missouri Wildflowers (http://mowildflowers.net/) for *Coreopsis* and Hamilton Native Outpost (https://www.hamiltonnativeoutpost.com/) for *Chamaecrista*. We lack data on the genetic variability present in the seed lots, but the original source of seeds for both species was <300 km from the study site. Specifically, the *Coreopsis* seeds were collected from wild plants in Newton County, MO (~275 km southeast of the study site) in 2016 or 2017. Details for *Chamaecrista* are less certain, but the likely original wild population was a prairie in Gentry County, MO (~150 km northeast of the study site).

*Coreopsis* seeds were not scarified, while *Chamaecrista* seeds were scarified by placing the seeds in a bag of coarse sand, followed by vigorous shaking and rubbing. Seeds of both species were then sowed into flats of autoclaved potting soil and placed into cold stratification for 4 weeks. Once the seedlings were large enough (approximately 5.0 cm tall for *Chamaecrista* and approximately 3.0 cm rosette diameter for *Coreopsis*), they were planted into groove tubes (98 ml, Stuewe and Sons GT51D) filled with their respective soil type (native whole, native AM fungi enhanced, uninoculated, see above). The *Chamaecrista* seedlings began to wilt and lose color, so they were treated twice with rhizobia (*Bradyrhizobium frederickii* and *Bradyrhizobium niftali*) purchased from Prairie Moon. A mixture of 1.23 ml of rhizobia with 1,000 ml of water was distributed evenly over the ~150 seedlings. After the treatments, seedlings revived and regained healthy coloration. The seedlings remained in groove tubes in the greenhouse for 9–10 weeks before they were transplanted into the field plots.

We lack data on survival from sown seeds to the seedlings that emerged in the flats. We also lack data on survival of seedlings from planting into the groove tubes until seedlings were transplanted into the field plots, although our notes suggest that survivorship was very high for these species.

#### 2.2.3 Field plots

Seedlings in the three treatments were planted into twice‐tilled field plots at the University of Kansas Field Station. The experimental site was historically tallgrass prairie; Kettle, Rich, Kindscher, Pittman, and Fu (2000) examined the General Land Office survey records from 1856 to 1860 and found the site was prairie with no woody vegetation recorded. From at least 1870–1970, the land was in agricultural use with both cropland and pasture (D. Kettle, personal communications). Hence, we refer to the soil as postagricultural. Cool‐season nonnative grasses have predominated for the last 50 years. Postagricultural soils such as these do have a microbial community with both mutualists and pathogens. However, AM fungal species in such degraded soils tend to be weedy and less likely to have positive effects on growth of prairie species (House & Bever, 2018; Koziol et al., 2018).

Plots were 1.5 m^2^ with 0.5 m aisles between plots within a treatment, and 2 m aisles between soil treatments. The native whole‐soil and uninoculated plots each received 75.71 L of soil in the center of each plot, which was then raked in and tilled one more time. The native AM fungal treatments received 3.63 L of AM fungal inoculum onto the tilled postagricultural soil (no autoclaved soil was added to plots in this treatment). Our study was part of a larger study examining the effects of soil types, plant species richness, and plant phylogenetic relationships on plant communities among 18 species across three families (396 plots). We focus here on the 136 plots that had seedlings of *Chamaecrista* or *Coreopsis*. The remaining 16 species are perennials and fitness studies thus require multiple years of data.

In each of these plots, 18 seedlings were planted per plot equidistant from each other. These plots included monocultures of *Chamaecrista* or *Coreopsis*, as well as plots where each of these species was planted in conjunction with other species. The latter included plantings of 2 species, 3 species, 6 species, and 18 species. Within the 2, 3, and 6 species plots, plots had plants of *Chamaecrista* or *Coreopsis* and also other species that were either phylogenetically similar (i.e., other species within the same family) or phylogenetically diverse (i.e., other species from different families). Phylogenetically similar species were from the Fabaceae for *Chamaecrista* and from the Asteraceae for *Coreopsis*. For the phylogenetically diverse treatment, other species were from Asteraceae or Poaceae for *Chamaecrista* or from Fabaceae or Poaceae for *Coreopsis*. The 18 species plots had 18 different plant species, with 6 each from Poaceae, Fabaceae, and Asteraceae. The within‐plot locations of the 18 species were randomized for all plots, with the locations of plants staying the same by block (1 sub‐block containing 3 soil treatments). The seedlings were planted in small holes between the dates of 30 May–13 June 2018 by sub‐block. Prior to planting the seedlings (29 May–5 June 2018), 50 seeds of each of 71 prairie species were sprinkled as evenly as possible across the plots, then raked in. No data were recorded for this study from plants germinating from added seed. We focused only on the planted seedlings, which were watered for establishment daily during the first week, every other day for the second week, and every third day for the third week. After 3 weeks, watering was discontinued. See Appendix 1 for experimental details.

### 2.3 Data collection

#### 2.3.1 Plant survival and reproduction

We collected data on survival and reproduction of *Coreopsis* and *Chamaecrista* by sub‐block, 4 weeks after seedlings were transplanted into the field plots (5–25 July 2018). We assessed if plants were present, and if they were flowering. If the plant was determined to be both present and flowering, we counted the number of flowers (defined to include flowers, buds, and seed heads/pods).

#### 2.3.2 Seed head collection (only *Coreopsis*)

We used the plant richness assigned to a plot to determine the number of plants to be sampled per plot (2 plants for mono‐, bi‐, and tricultures, 1 plant for 6 and 18 species plots). Within a plot, we randomly chose a plant and then collected a sample of mature seed heads where seeds had not dispersed (10% of the previously recorded flower number) on 2–3 August 2018. The harvested seed heads were collected from throughout the plant and placed in a paper envelope labeled with the plant and number of seed heads collected. The envelope was then dried and weighed (to nearest 0.001 g).

#### 2.3.3 Environmental data

Given that the effects of microbes on plants are often affected by environmental conditions, we determined the centimeters of precipitation at the KU Field Station from 1 January to 31 July 2018 using the CoCoRaHS network (all our data collection was finished in early August). Pooled soil samples from each treatment within each of the six sub‐blocks (18 samples total) were analyzed for soil abiotic properties and soil microbial composition. One 2 cm diameter by 20 cm deep core of soil was collected from six plots within each inocula treatment*subblock randomly chosen to represent the six different plant richness treatment (0, 1, 2, 3, 6, and 18 planted species). Soil from the plots was pooled into one sample which was partitioned into one part for analysis of abiotic properties and a second part for extraction of DNA for analysis of microbial composition. Soil texture, pH, organic matter, total C, total N, and total P were analyzed by Kansas State University Soil Testing Laboratory (Manhattan, KS).

#### 2.3.4 DNA extraction and sequencing processing

DNA was extracted from 0.25 g fresh soil following the manufacturer's instructions (DNeasy PowerSoil Kit; Qiagen), and bacterial, fungal, and AM fungal communities were sequenced. The bacterial primers (forward 515F, 5′‐GTGYCAGCMGCCGCGGTAA‐3′; reverse 806R 5′‐GGACTACNVGGGTWTCTAAT‐3′) targeting the V4 region of the 16S small subunit (SSU) rRNA (Apprill, McNally, Parsons, & Weber, 2015; Parada, Needham, & Fuhrman, 2016), fungal primers (forward fITS7, 5′‐GTGAGTCATCGAATCTTTG; reverse ITS4, 5′‐TCCTCCGCTTATTGATATGC‐3′) targeting the internal transcribed spacer (ITS) region (Ihrmark et al., 2012), and AM fungal primers (forward fLROR, 5′‐ACCCGCTGAACTTAAGC‐3′; reverse FLR2, 5′‐ TCGTTTAAAGCCATTACGTC‐3′) designed for the large subunit (LSU) region (House & Bever, 2018) were selected for polymerase chain reaction (PCR). PCRs were conducted in a final volume of 25 μl with 1 μl template DNA, 10.5 (fungi and AM fungal) or 13 (bacteria) μl ddH_2_O, 0.5 μl of forward and reverse primer, and 12.5 (fungi and AM fungi) or 10 (bacteria) μl of Master Mix Phusion (Thermo Fisher Scientific). The bacterial PCR program was as follows: 98°C for 30 s, 35 × (98°C for 10 s, 57°C for 30 s, and 72°C for 30 s), ending with 72°C for 2 min. The fungal PCR program was as follows: 94°C for 5 min; 35  ×  (94°C for 30 s; 57°C for 30 s and 72°C for 30 s); and 72°C for 7 min. The AM fungal PCR program was as follows: 94°C for 5 min, 35 × (94°C for 30 s, 48°C for 30 s, and 72°C for 45 s), ending with 72°C for 10 min. Four microliters of PCR product were checked on 1.5% (w/v) agarose gel to estimate the quality of PCR products. PCR products were barcoded using Nextera XT Index Kit v2 (Illumina) for indexing and purified using AMPure XP beads (Beckman Coulter) before sequencing. PCR product concentration was measured by Invitrogen Qubit 3.0 Fluorometer (Thermo Fisher Scientific). Adaptor ligation and sequencing were performed by Illumina MiSeq v3 PE300 Next‐Gen Sequencer in Genome Sequencing Core of University of Kansas. Sequences were submitted to the NCBI Sequence Read Archive (SRA) under the accession number PRJNA609011.

After sequencing, the primary analysis of raw FASTQ data was processed with the QIIME2 pipeline (Bolyen et al., 2019). Briefly, after demultiplexing and removing primers, sequences were quality filtered, trimmed, denoised, and merged using DADA2 (Callahan et al., 2016). Chimeric sequences were identified and removed via the consensus method in DADA2. The OTUs that only appeared 5 times or fewer across all samples were discarded to preclude inclusion of sequences from potential contamination or sequencing errors. Taxonomy was assigned to all ribosomal sequence variants in QIIME2 using a feature classifier trained with the SILVA 99% OTU database for bacteria (Quast et al., 2012) and the UNITE 99% database for fungi (Version 18.11.2018). Putative pathogens from the fungal community were categorized based on the FUNGuild database (Nguyen et al., 2016), and only OTUs with tropic mode as “Pathotroph” were used for the subsequent analysis. For AM fungal LSU amplicons, we excluded non‐AM fungal sequences by generating a phylogenetic tree that included a curated database base of AM fungal sequences (House et al., 2016; Krüger, Krüger, Walker, Stockinger, & Schüßler, 2012) and used sequences with *Mortierella elongata* as an out‐group (House & Bever, 2018). On average, 1,401 (398–2,078) quality 16S sequences, 8,490 (3,909–15,914) quality ITS sequences, and 73 (9–253) quality LSU sequences were obtained per sample, and OTU tables were rarefied to a sequencing depth of 398, 3,909 reads per sample for bacterial and fungal communities, respectively, prior to downstream analyses. For AM fungal LSU sequences, one sample was discarded when doing the analyses due to low amplifications, and we did not do the rarefication because of low reads.

### 2.4 Statistical analysis

Statistical analyses were conducted on two types of plant variables. First, we analyzed probability of survival and probability of flowering given survival; in both cases, we assumed a binomial distribution. Second, for plants that flowered, we analyzed number of flowers for each species, and average mass per seed head (only *Coreopsis*); these data were log‐transformed. We analyzed fitness (log‐transformed) as the product of three terms: the probability of plant survival, the probability of flowering if plants survived, and the average number of flowers produced per flowering plant. For all analyses, we used mixed models with soil treatment, plant richness, plot phylogeny, plant richness*plot phylogeny, and sub block as fixed effects, and plot phylogeny*sub block*treatment*plot and plot phylogeny*sub block*treatment as the random effects. These random effects account for the potential nonindependence of individual plants within the same plots. Analysis of survival for *Coreopsis* did not converge because of very high rates of survival in some treatments. All analyses were performed using SAS^®^ University software, with PROC GLIMMIX (probabilities of survival and flowering) and PROC MIXED (flower number, fitness, and average mass per seed head). We specifically tested for differences between each of the inocula treatment means and controlled for multiple comparisons using Tukey's adjustment. We present least square means and *SE*.

The Shannon–Wiener diversity index and the richness of bacterial, fungal, fungal pathogen, and mycorrhizal fungi communities for each sample were calculated using the diversity function in *vegan* package (Oksanen et al., 2016) in R (version 3.5.1). ANOVA analysis was conducted in SAS to test the statistical significance of Shannon and richness among different soil treatments. In order to test whether microbial communities were significantly different among soil inoculations, we performed permutational multivariate analysis (*adonis*) on the sequence density of OTUs. This was conducted with 1,000 permutations using a Euclidean distance matrix. We illustrate these differences in composition using principal component analysis (PCA). The principal component analyses of microbial communities were performed with the *factoextra* package (Kassambara & Mundt, 2017). As the major axes of variation were most strongly associated with spatial differences in the field, we chose to depict the first two PCA axes that are most sensitive to the inoculation treatment.

## 3 RESULTS

### 3.1 *Chamaecrista*

**FIGURE 2:**
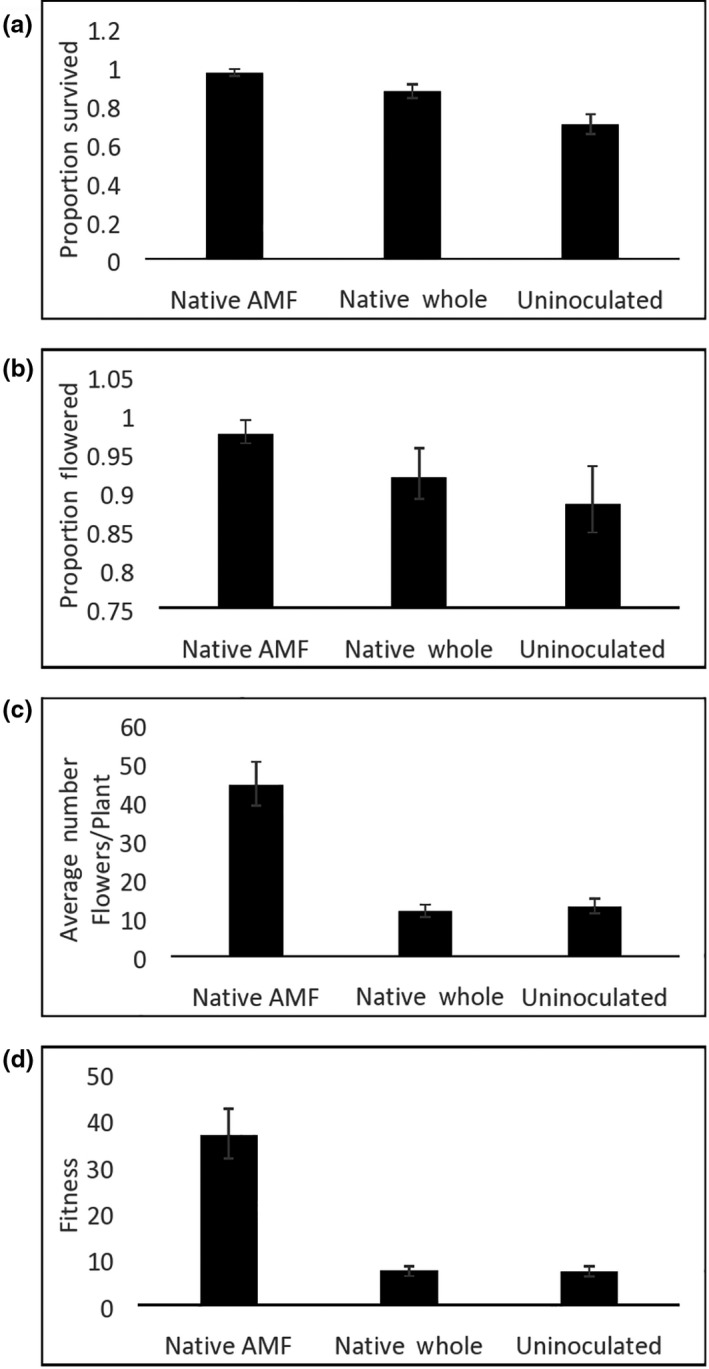
Survival and reproduction of *Chamaecrista* depending on treatment (native AM fungi enhanced, native whole prairie, and uninoculated). (a) Proportion of plants that survived; (b) proportion of surviving plants that flowered; (c) average number of flowers per flowering plant; and (d) fitness. Least square means and *SE* are shown

Plants receiving native AM fungi‐enhanced inocula had higher probability of survival, probability of flowering, and greater number of flowers per flowering plant compared to uninoculated plants, and higher survival and greater number of flowers per flowering plant compared to plants inoculated with native whole soil (Table 1 and Figure 2a–c). Plants inoculated with native whole soil had a higher probability of survival compared to uninoculated plants, but no significant differences were found for probability of a plant to flower, or in the number of flowers produced per flowering plant (Table 1 and Figure 2a–c). Fitness for *Chamaecrista* was highest in plants inoculated with native AM fungi‐enhanced inocula where plants produced on average approximately 5 times the number of flowers as plants in the other two treatments (Table 1 and Figure 2d).

**TABLE 1:** Analysis of probability of survival, probability of flowering, average number of flowers per plant, and fitness for *Chamaecrista* with comparisons between soil treatments

*Chamaecrista*	Survival			Flowering			# Flowers			Fitness		
	*df*	*F*	*p*	*df*	*F*	*p*	*df*	*F*	*p*	*df*	*F*	*p*
Treatment	2, 361	12.32	**<.0001**	2, 291	3.41	**.034**	2, 255	36.99	**<.0001**	2,361	55.59	**<.0001**
*AMF* versus* Uninoculated*	1, 361	22.27	**<.0001**	1, 291	6.73	**.02**	1, 255	45.6	**<.0001**	1,361	95.88	**<.0001**
*AMF* versus *Whole*	1, 361	6.48	**.023**	1, 291	3.89	.099	1, 255	58.74	**<.0001**	1,361	68.73	**<.0001**
*Whole* versus *Uninoculated*	1, 361	7.93	**.01**	1, 291	0.6	.88	1, 255	0.28	1.00	1,361	2.09	.149
Plant richness	1, 361	1.52	.218	1, 291	0.63	.429	1, 255	0.33	.565	1,361	0.25	.62
Sub block	5, 361	1.55	.173	5, 291	2.03	.075	5, 255	6.25	**<.0001**	5,361	7.25	**<.0001**
Plot Phylo	1, 15	0.25	.628	1, 15	0.33	.577	1, 15	0.02	.89	1,15	0.08	.781
Plant richness*Plot Phylo	1, 361	0.59	.445	1, 291	0.09	.766	1, 255	0.07	.793	1,361	0.03	.872

Note

Specific contrasts shown in italics. See text and Appendix 1 for full explanation of treatments, plant richness, and the phylogenetic (= phylo) treatment. See TableA1 for covariance estimates. Bold values are statistically significant *p*‐value (*p* < .05)

### 3.2 *Coreopsis*

**FIGURE 3:**
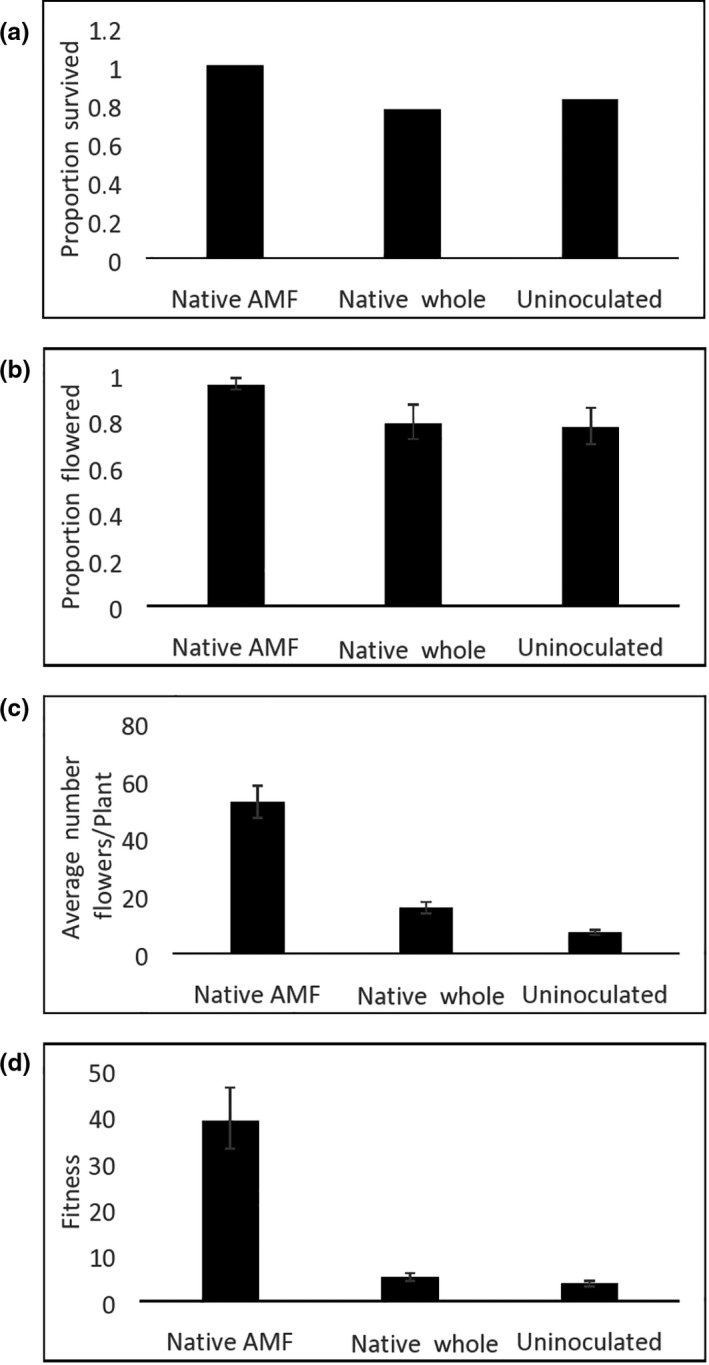
Survival and reproduction of *Coreopsis* depending on treatment (native AM fungi enhanced, native whole prairie, and uninoculated). (a) Proportion of plants that survived; (b) proportion of surviving plants that flowered; (c) average number of flowers per flowering plant; and (d) fitness. Least square means and *SE* are shown, except for survival (see text)

The analyses for probability of survival were not computable due to the limited variation in the native AM fungal inocula treatment (all plants survived). However, a simple comparison of the percent survival of plants across the treatments suggests that plants inoculated with native AM fungi‐enhanced inocula performed the best (Figure 3a). Plants with native AM fungi‐enhanced inocula had a higher probability of flowering and flowering plants produced greater numbers of flowers than uninoculated plants (Table 2 and Figure 3b,c). Native AM fungi‐enhanced inoculated plants also produced greater numbers of flowers per flowering plant when compared with plants inoculated with native whole soil, but were not significantly different (*p* = .06) in their probability to flower (Table 2 and Figure 3b,c). Plants inoculated with native whole soil flowered with similar probabilities, but produced a greater number of flowers per plant compared to uninoculated plants (Table 2 and Figure 3b,c). There were no significant differences among treatments in average mass per seed head (Table 2). Fitness for *Coreopsis* was highest in plants inoculated with native AM fungi‐enhanced soil, approximately 10 times greater than plants in uninoculated plots, and 7 times greater than those in the native whole‐soil treatment (Table 2 and Figure 3d).

**TABLE 2:** Analysis of probability of survival, probability of flowering, average number of flowers per plant, fitness, and average mass per seed head (SH) for *Coreopsis* with comparisons between soil treatments

*Coreopsis*	Survival			Flowering			# Flowers			Fitness			Avg Mass/SH		
	*df*	*F*	*p*	*df*	*F*	*p*	*df*	*F*	*p*	*df*	*F*	*p*	*df*	*F*	*p*
Treatment	—	—	—	2, 10	4.21	**.047**	2, 239	90.7	**<.0001**	2, 356	72.45	**<.0001**	2, 50	2.39	.102
*AMF* versus*. Uninoculated*	—	—	—	1, 10	7.19	**.046**	1, 239	172.7	**<.0001**	1, 356	122.2	**<.0001**	1, 50	4.34	.085
*AMF* versus*. Whole*	—	—	—	1, 10	6.39	**.06**	1, 239	63.14	**<.0001**	1, 356	94.45	**<.0001**	1, 50	0.04	1.00
*Whole* versus*. Uninoculated*	—	—	—	1, 10	0.03	1.00	1, 239	22.64	**<.0001**	1, 356	2.47	.117	1, 50	3.4	.142
Plant richness	—	—	—	1, 300	4.59	**.033**	1, 239	0.91	.342	1, 356	0.31	.577	1, 50	0.01	.915
Sub block	—	—	—	5, 10	1.27	.348	5, 239	9.27	**<.0001**	5, 356	6.78	**<.0001**	5, 50	1.03	.411
Plot Phylo	—	—	—	—	—	—	1, 11	0.05	.819	1, 11	0.02	.898	1, 9	0.95	.354
Plant richness*Plot Phylo	—	—	—	—	—	—	1, 239	0.66	.419	1, 356	0.61	.435	1, 50	1.91	.173

Note

Specific contrasts shown in italics. The presence of a “—” in a cell in the table refers to models that did not converge (survival) or features that were not included in a particular model (flowering). See text and Appendix 1 for full explanation of treatments, plant richness, and the phylogenetic (= phylo) treatment. See TableA2 for covariance estimates. Bold values are statistically significant *p*‐value (*p* < .06)

### 3.3 Environmental data

The 2018 value (34.87 cm) for 1 January–31 July rainfall was the lowest for the same months in the 10‐year period of 2010–2019 (average 56.36 cm). There were no significant differences in soil properties among three inoculation treatments. The soil on average contains 12% sand, 58% silt, and 30% clay, with a pH of 5.90. There was 4.94% organic matter, 2.89% total C, 0.24% total N, and 353 mg/kg total P.

### 3.4 Microbial communities among different soil inoculations

**FIGURE 4:**
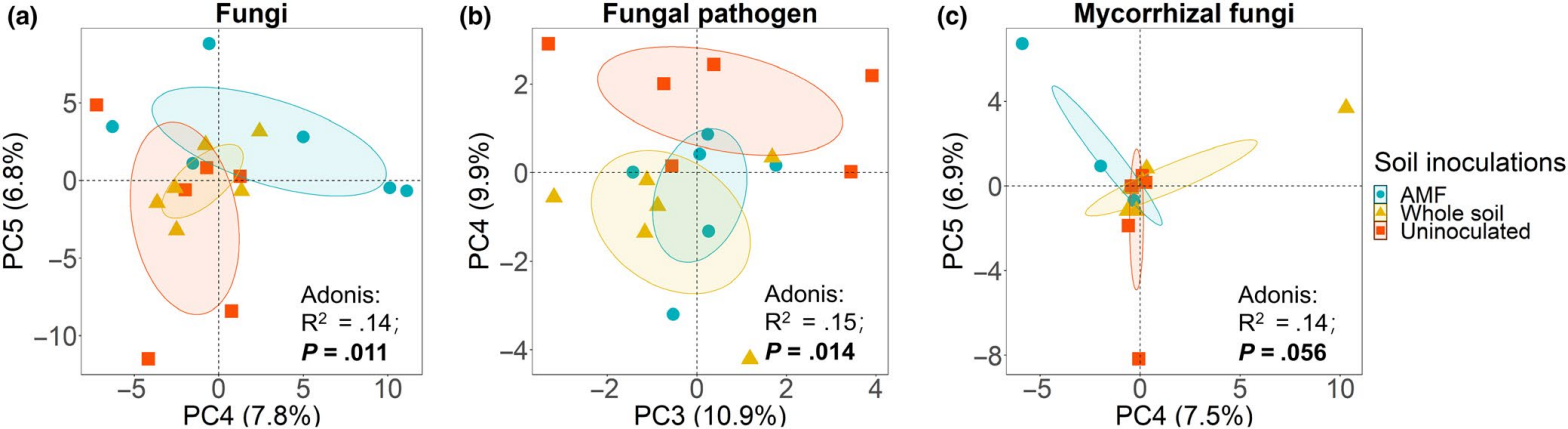
Principal component analysis (PCA) ordinations of fungal (a), fungal pathogen (b), and AM fungal (c) communities in three soil inoculations. The two most sensitive axes are shown in the plots. Each dot corresponds to an individual sample, colored by soil treatments. “AMF”, “whole soil,” and “uninoculated” represent native AM fungal enhanced soil, native whole soil, and uninoculated postagricultural soil, respectively

**FIGURE A2:**
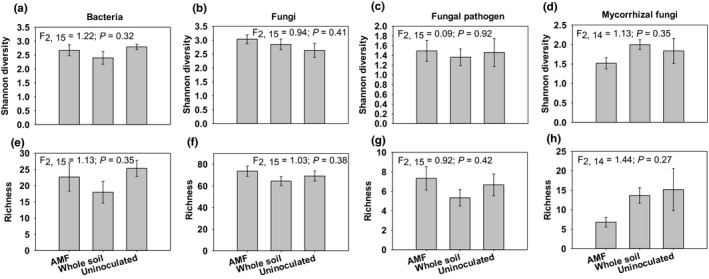
Microbe communities. Shannon diversity index and richness of bacterial (a, e), fungal (b, f), fungal pathogen (c, g), and AM fungal (d, h) communities in three soil inoculations. The statistical significance of microbial community among different soil treatments is given at the top of each plot. “AMF,” “whole soil,” and “uninoculated” represent native AM fungi‐enhanced soil, native whole soil, and uninoculated postagricultural soil, respectively

**FIGURE A3:**
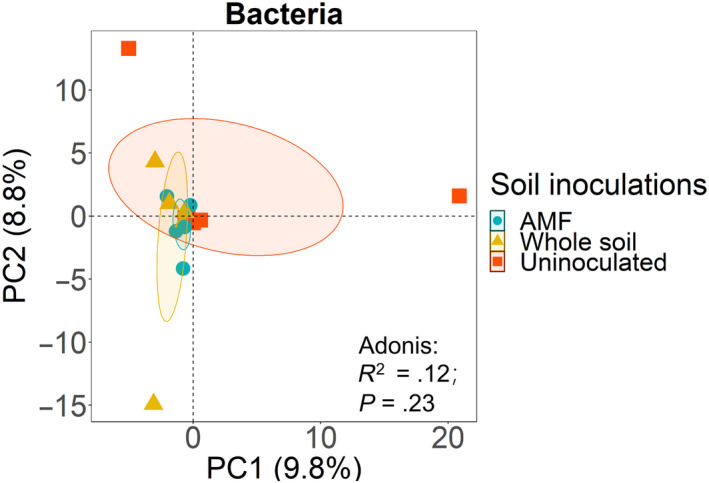
Bacterial communities. Principal component analysis (PCA) ordinations of bacterial communities in three soil inoculations. Each dot corresponds to an individual sample, colored by soil treatments. “AMF,” “whole soil,” and “uninoculated” represent native AM fungal enhanced soil, native whole soil, and uninoculated postagricultural soil, respectively

For soil microbial communities (bacteria, fungi, pathogenic fungi, and AM fungi), no significant differences were observed among the three soil inoculations as to Shannon diversity and OTU richness (Figure A2). The PERMANOVA of bacterial composition did not show differences between inocula treatments (Figure A3, *p* = .23); however, we did observe significant differences in composition with inoculation for fungi overall (inoculation treatment pseudo *R* ^2^ = .14, *p* = .011), for pathogenic fungi (inoculation treatment pseudo *R* ^2^ = .15, *p* = .014), and AM fungi (inoculation treatment pseudo *R* ^2^ = .14, *p* = .056). Differences in composition are illustrated using principle component axes that were most sensitive to the inoculation treatment (Figure 4).

## 4 DISCUSSION

### 4.1 Response to arbuscular mycorrhizal fungi

For both annual plant species, the addition of native AM fungi had major positive effects on plant survival, reproduction, and fitness relative to the uninoculated treatment. Additionally, we saw that the native whole‐soil treatment had either no effect or a positive effect on plant performance, which suggests that soil mutualists were an important component of the whole prairie soil. The strong positive effects of native AM fungi on annual species in our work are in contrast to other studies that show early successional species to have relatively little response to AM fungal additions or sensitivity to AM fungal composition (Cheeke et al., 2019; Koziol & Bever, 2015, 2016). Major positive effects of native AM fungal additions (or native whole‐soil additions) have been primarily found in studies of longer living perennial plants (House & Bever, 2019; Koziol & Bever, 2017; Koziol et al., 2018; Middleton & Bever, 2012; Middleton et al., 2015). Our results are consistent, however, with a recent mesocosm study which found a native annual grass showing strong benefits of locally adapted native AM fungi (Bauer, Koziol, & Bever, 2020). A consistent aspect of our study, and the previous mesocosm study, is that they were performed in an early successional competitive context. As succession proceeds and later successional species are established, it may well be that these same annual species that once benefited from native AM fungi would then be competitively suppressed because of the differential advantage of later successional plant species to native AM fungi.

The effect of AM fungi on plant performance can also depend on the environmental context (Hoeksema et al., 2010, 2018). Long‐term nutrient addition experiments at this site identify that the plant community can be colimited by N and P (B. Foster, personal communication). The fact that native AM fungi led to enhanced annual plant performance is consistent with the fact that the study was done in degraded soil, likely lacking in nutrients like phosphorous. Moreover, there was a severe drought for the duration of this study which may have increased the dependence of plants on AM fungi.

### 4.2 Response to soil pathogens

We found that *Coreopsis* and *Chamaecrista* performed better with native AM fungi‐enhanced inocula than with native whole‐soil inocula. Given that the native whole‐soil inocula likely include both native AM fungi and native soil pathogens, it appears that these early successional annuals were vulnerable to native pathogens. This result is consistent with previous work showing negative effects of native whole soil on early successional plant species (Middleton & Bever, 2012). Moreover, given that pathogens often have host‐specific effects that can drive feedbacks (Bever, Platt, & Morton, 2012; Crawford et al., 2019), our results are consistent with evidence of strong negative plant–soil feedbacks on early successional plant species (Bauer et al., 2015). The ratio of host‐specific pathogens to mutualists in the native whole‐soil type likely largely depends on the presence, and abundance of plant species at the location the soil was taken from. We note that *Chamaecrista* was present at the source of the remnant prairie soil, but *Coreopsis* was not (T. Michaels, personal communications). Our use of a soil with history of occupancy with *Chamaecrista* could have contributed to this plant species' poorer performance in native whole soil relative to uninoculated soil for flower number. In contrast, *Coreopsis* performed significantly better for flower number in native whole soil than in uninoculated soil, suggesting that the benefits of native AM fungi outweighed the negative impacts of pathogens (Table 2 and Figure 3a–c). Importantly, our evidence of pathogen impacts in this study comes from a single season of growth following tillage of the field plots. It is likely that subsequent years of growth in these same plots will result in even stronger negative impacts due to accumulation of host‐specific pathogens (Crawford et al., 2019).

The decreased performance in native whole soil compared to soil enhanced with native AM fungi is consistent with the effects of native pathogens. However, the AM fungal community in the native AM fungi‐enhanced soil is also different from that in the native whole soil, as only a subset of the AM fungi present at the prairie from which the soil was derived were successfully cultured. Further, these AM fungal cultures were then supplemented by cultures of AM fungi derived from other prairies. This change in composition of native AM fungi is confounded with the difference in native pathogens in the two treatments and thus creates a second plausible hypothesis for the different response of the two plant species to the native whole soil compared to native AM fungi‐enhanced soil. Environmental sequencing of our experimental plots cannot differentiate the pathogen and AM fungal mechanisms, as it identifies that the inoculation treatments were successful in changing both fungal pathogen and AM fungal composition. Further, we do not know whether the densities of AM fungal spores were similar in the AM fungi‐enhanced soil and in the native whole soil. While particular AM fungal species do have differential effects on plant species growth rates, early successional species tend to be less sensitive to different AM fungal species than late successional species (Cheeke et al., 2019; Koziol & Bever, 2016). We also note that late successional plant species responded similarly to the native AM fungal enhanced soil and the native whole‐soil inocula (Wagner, unpublished data). This result suggests that differences in AM fungal composition are unlikely to be responsible for the differences between the native AM fungi enhanced and native whole‐soil treatments.

### 4.3 Plant life cycles and importance of soil inocula in prairie restorations

Associations between soil microbes and plant roots likely developed early in plant life cycles given that seedlings were transplanted into the experimental soil treatments. It is therefore perhaps not surprising that effects of the soil treatments were seen at multiple points in the life cycles of the two species, with average number of flowers produced per flowering plant being most affected by the treatments applied. This late stage of the life cycle may have the greatest differences among soil types because effects of the treatments on plant size may have started out small at the start of the plant's life but became more substantial as the plants grew and flowered.

Our study was unusual in examining effects of soil treatments on both plant survival and reproduction under field conditions. Future studies should address other factors affecting fitness, such as possible maternal effects (Alexander et al., 2014) or treatment effects on seedlings prior to transplantation into the field site. Although we were unable to obtain seed head mass for *Chamaecrista,* we did have such data for *Coreopsis*. We found no differences across the treatments for average mass per seed head. This could suggest that the number of seeds produced per flower and mass per seed in each soil type is the same even though the number of flowers produced by plants in the three treatments was different. However, there are multiple factors that could be causing the result that we saw, such as the seeds within each seed head could be fewer in number but bigger. We did not count the number of individual seeds per seed head across the treatments, nor did we examine seed viability. Thus, we do not know if soil treatment had any effect on plant fitness later in the life cycle than number of flowers produced.

The effects of soil microbes across the plant life cycle are of potential relevance to prairie restoration efforts. Our results suggest that for early successional prairie annuals, native AM fungi enhancement of soil may be beneficial for conversion of disturbed, postagricultural land into prairie restoration sites. It is intriguing, however, that Koide and Dickie (2002) found that as plant densities increase (as is typical of annual prairie plants in early years of a restoration), the positive effects of AM fungi often decline. Further, soil pathogens that infect these annuals would be expected to accumulate. Given that inoculation of prairie restorations with native soils (with a developed microbial community) particularly benefit late successional prairie species, the overall effects of native soil microbe additions are likely an acceleration of succession in prairie restorations (Koziol et al., 2018; Lubin et al., 2019; Middleton & Bever, 2012). Adding native AM fungi and other microbes to the soil is thus a promising approach for more effective conversion of damaged landscapes into ecosystems that resemble the diverse prairies that were once so abundant in center of North America.

## CONFLICT OF INTEREST

None declared.

## AUTHOR CONTRIBUTION

**Hannah S. Reynolds:** Conceptualization (equal); Data curation (lead); Formal analysis (equal); Investigation (equal); Methodology (equal); Visualization (equal); Writing‐original draft (equal); Writing‐review & editing (equal). **Rebekah Wagner:** Conceptualization (equal); Data curation (supporting); Investigation (equal); Methodology (equal). **Guangzhou Wang:** Conceptualization (supporting); Formal analysis (supporting); Investigation (supporting); Methodology (supporting); Visualization (supporting); Writing‐review & editing (supporting). **Haley M. Burrill:** Conceptualization (supporting); Formal analysis (supporting); Investigation (supporting); Methodology (supporting); Visualization (supporting); Writing‐review & editing (supporting). **James D. Bever:** Conceptualization (equal); Formal analysis (equal); Funding acquisition (lead); Investigation (equal); Methodology (equal); Project administration (lead); Resources (lead); Supervision (supporting); Visualization (supporting); Writing‐original draft (supporting); Writing‐review & editing (equal). **Helen M. Alexander:** Conceptualization (equal); Funding acquisition (supporting); Investigation (supporting); Methodology (equal); Supervision (lead); Writing‐original draft (equal); Writing‐review & editing (equal).

## REFERENCES

House, G. L., & Bever, J. D. (2019). Biochar soil amendments in prairie restorations do not interfere with the benefits provided by arbuscular mycorrhizal fungi. Restoration Ecology. https://doi.org/10.1111/rec.12924

Koziol, L., Crews, T. E., & Bever, J. D. (2019). Benefits of native mycorrhizal amendments to perennial agroecosystems increases with field inoculation density. Agronomy, 9, 353. https://doi.org/10.3390/agronomy9070353

Koziol, L., & Bever, J. D. (2017). The missing link in grassland restoration: arbuscular mycorrhizal fungi inoculation increases plant diversity and accelerates succession. Journal of Applied Ecology, 54, 1301–1309.

Wang, G., Schultz, P., Tipton, A., Zhang, J., Zhang, F., & Bever, J. D. (2019). Microbiome mediation of positive plant productivity‐diversity relationships in late successional grassland species. Ecology Letters, 22, 1221–1232. https://doi.org/10.1111/ele.13273

## Figures and Tables

**FIGURE A1:**
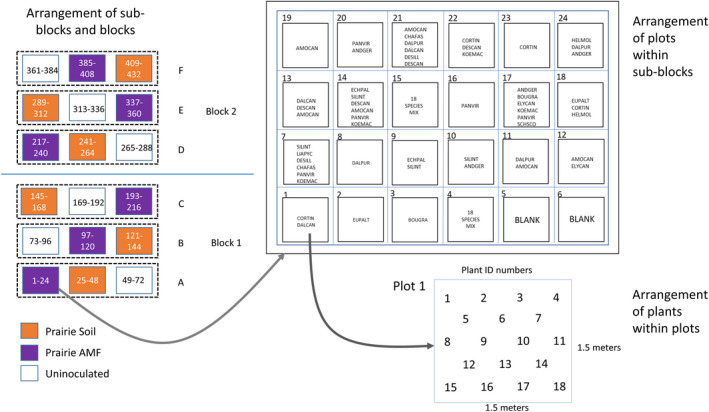
Experimental design. Eighteen different prairie plant species were planted into plots that included a range of plant species richness (1, 2, 3, 6, and 18) and phylogenetic dispersion levels (within family or between plant family). Each plot included eighteen individual seedlings. Plots were arranged in groupings of twenty‐four plots, each of which received the same inoculation treatment. The three inoculation treatments were native prairie whole soil, native prairie AM fungi‐enhanced soil, and an uninoculated control. Groupings of 24 plots of each of the treatments were arranged in spatial sub‐blocks. Three sub‐blocks were grouped into complete blocks, within which each plant species was represented equally within all treatment combinations

## Data Availability

Data available from the Dryad Digital Repository: https://doi.org/10.5061/dryad.47d7wm39h

## Appendix 1

### ADDITIONAL DESCRIPTION OF EXPERIMENTAL DESIGN

#### Details of experimental design

Our study takes place within the context of a larger experiment testing soil microbial mediation of ecosystem responses to variation in plant diversity. This experiment manipulates plant species richness, plant phylogenetic dispersion, and soil inoculation treatments in full factorial combinations. It uses 18 native prairie plant species, each of which is represented equally in all treatment combinations. These 18 plant species include species from three plant families: six grasses, six legumes, and six composites. Our study focuses on the demographic response of the two annual plant species, and the rest are perennial. The full design and the experimental layout are described in Figure A1. While 18 seedlings were planted into each plot, a given plant species would be represented in plots in which it was present as anywhere from 1 to 18 seedlings depending on the species richness treatment (1, 2, 3, 6, 18 species per plot).

#### Soil collection and source of inocula

Native prairie field soil was collected from a previously unplowed section of Anderson County Prairie Preserve, located approximately 97 km south of the field site. The site was available for soil harvest because of a scheduled expansion of Kansas state highway 59. Soil was collected from an area approximately 17 m × 46 m. The top 5–7 cm was initially removed to reduce seed input. The next 15 cm was tilled, collected, and transported to the field site for inocula.

Our prairie AM fungal inocula was composed of a mixture of individual AM fungal cultures isolated from the Anderson County Prairie Preserve and other tallgrass prairies. While we had many cultures of AM fungi derived from unplowed sections of the Anderson County Prairie Preserve, assembling a sufficient volumeof prairie AM fungal inocula required pooling AM fungal cultures the Bever/Schultz Lab had isolated from unplowed prairies from other regions. The majority of the inocula came from the Anderson County Prairie Preserve and three other unplowed prairies within 129 km of our field site, and the remaining came from sites further east (Indiana). All of these isolates have been used in previous publications on impacts of native AM fungi in plant growth (e.g., House & Bever, 2019; Koziol & Bever, 2017; Koziol et al., 2019; Wang et al., 2019). While not all of our isolates have been identified, we know that our mixtures included representative AM fungi from *Scutellospora*, *Gigaspora*, *Claroideoglomus*, *Rhizophagus*, *Entrophospora*, *Acaulospora*, *Glomus*, and *Paraglomus* genera.

**TABLE A1:** Estimates of the covariance components from the analysis of the mixed models for *Chamaecrista*

Covariance parameter estimates	Estimate	*SE*
Survival		
Plot phylo*sub block*treatment	0	—
Plot phylo*sub block*treatment*plot	2.86E‐06	4.92E‐06
Residual	—	—
Flowering		
Plot phylo*sub block*treatment	0	—
Plot phylo*sub block*treatment*plot	9.97E‐06	0.000011
Residual	—	—
Flower number		
Plot phylo*sub block*treatment	0	—
Plot phylo*sub block*treatment*plot	2.46E‐06	—
Residual	1.0562	—
Fitness		
Plot phylo*sub block*treatment	0	—
Plot phylo*sub block*treatment*plot	1.32E‐06	—
Residual	1.8512	—

**TABLE A2:** Estimates of the covariance components from the analysis of the mixed models for *Coreopsis*

Covariance parameter estimates	Estimate	*SE*
Survival		
—	—	—
—	—	—
Residual	—	—
Flowering		
Sub block*treatment	0.6095	0.5376
Sub block*treatment*plot	0	—
Residual	—	—
Flower number		
Plot phylo*sub block*treatment	0.02183	—
Plot phylo*sub block*treatment*plot	1.12E‐06	—
Residual	0.6915	—
Fitness		
Plot phylo*sub block*treatment	0.07566	—
Plot phylo*sub block*treatment*plot	3.14E‐06	—
Residual	1.8377	—
